# Urinary Tract Infections among Indonesian Pregnant Women and Its Susceptibility Pattern

**DOI:** 10.1155/2020/9681632

**Published:** 2020-04-21

**Authors:** Yeva Rosana, Dwiana Ocviyanti, Melissa Halim, Friza Yossy Harlinda, Rahmah Amran, Wafridha Akbar, Matthew Billy, Syadza Rhizky Putri Akhmad

**Affiliations:** ^1^Department of Microbiology, Faculty of Medicine Universitas Indonesia, Ciptomangunkusumo Hospital, Jakarta, Indonesia; ^2^Department of Obstetrics and Gynecology, Faculty of Medicine Universitas Indonesia, Ciptomangunkusumo Hospital, Jakarta, Indonesia; ^3^Faculty of Medicine Universitas Indonesia, Indonesia

## Abstract

Pregnant women are usually at risk of urinary tract infections (UTIs) such as asymptomatic bacteriuria. In the current multidrug-resistance era, appropriate diagnosis and treatment should be provided to avoid complications in pregnant women in developing countries, which have limited facilities, such as Indonesia. The aim of this study was to evaluate in vitro susceptibility tests. Urinary isolates were collected from 715 pregnant women who visited eight Community Health Centers in Jakarta, Indonesia, between 2015 and 2017. We identified bacterial uropathogens from samples that were positive for nitrite/leukocyte esterase (LE), using two types of VITEK cards. Since noncompliance among patients is a major problem, fosfomycin-trometamol 3 g single-dose sachets were given to the patients, and the side effects of the medication and neonatal outcomes were reported. Asymptomatic bacteriuria was found in 10.5% of the 715 pregnant women. *Escherichia coli* was the most common etiological factor (26.7%), followed by *Klebsiella pneumoniae* (20%), *Streptococcus agalactiae* (9.3%), *Enterobacter cloacae* (5.3%), *Enterococcus faecalis* (5.3%), *Staphylococcus saprophyticus* (4%), *Acinetobacter baumannii* (4%), and others. Out of 76 pregnant women who took fosfomycin-trometamol, two complained of diarrhea that subsided without medication and fever that responded to paracetamol. Neonatal outcomes showed 100% full-term and normal-weight babies. *E. coli*, including extended-spectrum beta-lactamase- (ESBL-) producing *E. coli*, was 100% susceptible to fosfomycin. Nitrite/LE test results are often used as evidence for empiric antibiotic administration for treating asymptomatic bacteriuria in pregnancy, but the diagnosis should be confirmed using culture tests. Based on in vitro susceptibility patterns and medication outcomes, fosfomycin-trometamol single dose could be administered to noncompliant UTI patients, including pregnant women.

## 1. Introduction

Pregnant women form a part of the group at risk of urinary tract infections (UTIs). The prevalence of UTIs in pregnant women is approximately 2%–10% [1, 2]. Their reduced immunity appears to encourage the growth of both commensal and noncommensal microorganisms. UTI in pregnant women is frequently manifested as asymptomatic bacteriuria [2–4]. Asymptomatic bacteriuria in pregnancy poses a significant risk because untreated cases have a high likelihood (up to 40%) of progressing to acute pyelonephritis that can cause morbidity and even death of the mother and fetus [3]. Treatment should be provided immediately to prevent perinatal complications such as bacteremia, premature birth, and low birth weight [5, 6]. The choice of therapy is generally based on common pathogens encountered, susceptibility patterns, evidence, clinician consensus, antimicrobial stewardship principles, formulary availability, and antimicrobial costs [7, 8].

Fosfomycin-trometamol is recommended as one of the first-line agents for treating uncomplicated UTIs in the latest guidelines endorsed by the Infectious Diseases Society of America (IDSA), European Association of Urology (EAU), and the European Society for Clinical Microbiology and Infectious Diseases (ESCMID) [9, 10]. Since 2005, fosfomycin-trometamol has obtained marketing authorization in Indonesia and is being used widely in treating uncomplicated UTIs. The Indonesian Society of Obstetrics and Gynecology has recommended fosfomycin-trometamol as a safe treatment option for UTIs in pregnant women since 2015 under the National Guidelines for Medical Services. Single-dose treatment of fosfomycin-trometamol can help address the noncompliance issue among patients in developing countries, thus providing healing effects and preventing bacterial resistance. The appropriate antimicrobial agent for single-dose treatment should have a broad spectrum against both Gram-negative and Gram-positive uropathogens (low MICs), which should be proven using in vitro antimicrobial susceptibility tests [11]. Besides fosfomycin-trometamol, ampicillin-sulbactam is an antibiotic that is often used for treating pregnant women with UTIs in Indonesia [12].

Positive results on nitrite/leukocyte esterase (LE) tests are frequently used as evidence for empirical antibiotic administration for asymptomatic bacteriuria in Indonesia to avoid several complications including preterm labor, low birth weight, preeclampsia, hypertension, renal failure, and intrauterine fetal death. Generally, no evaluation was done for susceptibility pattern using culture and in vitro antimicrobial susceptibility tests. This strategy should be evaluated and reconsidered, particularly in the current multidrug-resistance era.

This study evaluated the diagnostic test that is usually carried out before starting empiric therapy. The evaluation of the effectiveness of fosfomycin-trometamol 3 g single dose, given to pregnant women, was carried out by observing clinical outcomes in mothers and neonates and an in vitro susceptibility test. The susceptibility patterns of relevant microorganisms to fosfomycin and other antibiotics including ampicillin-sulbactam were then compared.

## 2. Materials and Methods

### 2.1. Specimen Collection

Urinary isolates were collected from 715 pregnant women, who showed no symptoms of UTI and had visited one of the eight Community Health Centers in Jakarta, located in districts of Makassar, Pulogadung, Cakung, Pasar Rebo, Duren Sawit, Kramat Jati, Ciracas, and Matraman. The study was carried out from 2015 to 2017. Pregnant women aged between 14 and 35 years were included in the study. No urinary symptoms (e.g., UTI and vaginitis) were reported in the four weeks before this episode. Patients with recurrent UTIs and concomitant or prophylactic antimicrobial treatment for any reason seven days within the start of the study were excluded. Urine isolates were obtained from a clean-catch midstream urine sample. This study was approved by the ethical committee of the hospital and the Faculty of Medicine Universitas Indonesia (no. 896/UN2.F1/ETIK/2014), and all patients gave their written consent before being enrolled to the study.

### 2.2. Urine Analysis, Fosfomycin-Trometamol Therapy, and Culture

All urine isolates were tested for nitrite/leukocyte esterase (LE), and only the patients who tested positive for nitrite/LE were tested by urine culture and were given the option to choose a fosfomycin-trometamol 3 g single-dose sachet from this study or ampicillin-sulbactam from the Community Health Centers. Further, only the bacterial colonies from patients whose urine culture showed a colony count range greater than 100,000 CFU/mL (asymptomatic bacteriuria group) were tested for antibiotic susceptibility. The urine was cultured at the Clinical Microbiology Laboratory, Faculty of Medicine Universitas Indonesia. Before culturing, the urine sample was mixed well and evaluated using Gram stain. Using a sterile calibrated loop measuring 0.001 mL, urine was streaked on blood and MacConkey agar plates, followed by incubation at 35–37°C for 18–24 h. Patients whose samples showed a colony count range of ≥10^5^ CFU/mL on the initial urine culture were placed in the asymptomatic bacteriuria group. Urine isolates were identified using two types of VITEK cards: GN and GP cards used for the identification of Gram-negative bacilli and Gram-positive bacilli (primarily cocci), respectively (BioMérieux, Charbonnieres-les-Bains, France). The VITEK system is an automated system using kinetic analysis by reading each test every 15 min. The optical system combines a multichannel fluorimeter and photometer readings to record fluorescence, turbidity, and colorimetric signals. Colonies of a pure culture were taken and placed in a test tube containing 3.0 mL sterile saline (0.45%–0.50% NaCl, pH 4.5–7.0) to prepare a suspension. Suspension turbidity was adjusted to 0.5 McFarland, which was comparable to 1.5 × 10^8^ CFU/mL. Test tubes containing the microorganism suspension were placed in a special rack (cassette), and the identification card was placed in the neighboring slot while inserting the transfer tube into the corresponding suspension tube. The rack or cassette containing the test tubes and the identification card were inserted into the machine. The filled cassette was manually placed in a vacuum chamber station. After the vacuum was applied and air was reintroduced into the station, the microorganism suspension was forced through the transfer tube into the microchannels, filling all the test wells. The cassette was incubated at 35.5°C ± 1.0°C. Each card was removed from the carousel incubator once every 15 minutes, transported to the optical system for reaction readings, and then returned to the incubator until the next reading. The identification results appeared on the monitor screen after 3–7 h.

### 2.3. Antibiotic Susceptibility Testing

The susceptibility patterns of Gram-negative bacilli to 13 antibiotics were determined by VITEK AST-GN316 (BioMérieux), namely, to fosfomycin, ampicillin, ampicillin-sulbactam, gentamicin, cefalotin, trimethoprim/sulfamethoxazole (TMP/SMX), cefepime, cefotaxime, ceftazidime, ciprofloxacin, levofloxacin, meropenem, and piperacillin-tazobactam. VITEK AST-GP67 (BioMérieux) was used to determine the susceptibility of Gram-positive cocci to 16 antibiotics, namely, ampicillin, ampicillin-sulbactam, benzylpenicillin, ceftriaxone, clindamycin, ciprofloxacin, erythromycin, gentamicin, levofloxacin, linezolid, moxifloxacin, nitrofurantoin, tetracycline, tigecycline, TMP/SMX, and vancomycin. Susceptibility of Gram-negative bacilli and Gram-positive cocci to fosfomycin was also tested. The MICs break-point sensitivity test followed the Clinical and Laboratory Standards Institute (CLSI) guidelines [13]. Selecting antibiotics was based on availability on AST-GN316 and AST-GP67 panels, including those for first-line antibiotics commonly prescribed to treat bacterial urinary tract infections in pregnancy.

## 3. Results

### 3.1. Urine Analysis and Fosfomycin-Trometamol Therapy

Positive findings on the nitrite/LE test using dipstick were obtained for 135 (18.9%) out of 715 pregnant women. In this study, 76 patients who had positive results chose fosfomycin-trometamol over ampicillin-sulbactam (Table 1). The drug was meant to be dissolved in 100 mL water before consumption. Out of the 76 pregnant women who took the fosfomycin-trometamol 3 g single-dose sachet, two women complained of side effects consisting of diarrhea that subsided without medication and fever that responded to paracetamol, respectively.

### 3.2. Culture and Identification

**Figure 1:**
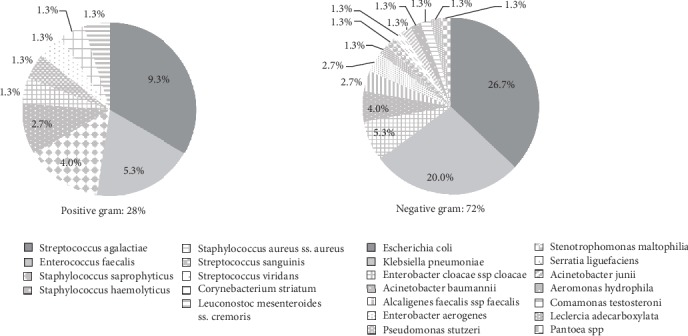
The pattern of bacteria found in pregnant women with asymptomatic bacteriuria in Jakarta, Indonesia.

Asymptomatic bacteriuria was confirmed by a colony count range of ≥100,000 CFU/mL on the initial urine culture. In this study, 73 (10.2%) of the 715 pregnant women had asymptomatic bacteriuria. Of the 73 pregnant women, the samples of 71 (97.3%) yielded single bacterial isolates while those of 2 (2.7%) yielded mixed bacterial isolates, giving a total of 75 isolates. Overall, the mixed bacterial isolates were found in the urine of two pregnant women in their third trimester (Table 1). Among the urine isolates, 21 (28%) were Gram positive and 54 (72%) were Gram negative. *Escherichia coli* was found to be the most common isolate (26.7%), followed by *Klebsiella pneumoniae* (20%), *Streptococcus agalactiae* (9.3%), *Enterobacter cloacae* (5.3%), *Enterococcus faecalis* (5.3%), *Staphylococcus saprophyticus* (4%), *Acinetobacter baumannii* (4%), *Alcaligenes faecalis* (2.7%), *Enterobacter aerogenes* (2.7%), *Staphylococcus haemolyticus* (2.7%), and others (Figure 1). Gram-negative bacteria were found more frequently than Gram-positive bacteria in the samples, particularly *E. coli* and *K. pneumoniae*.

### 3.3. Susceptibility to Antibiotics

**Figure 2:**
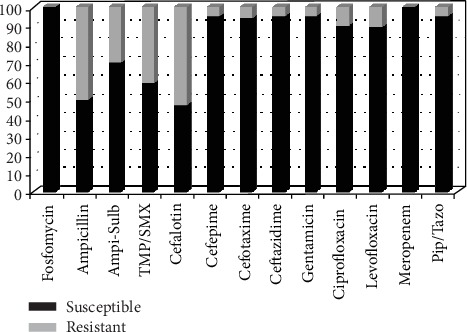
Percentage of *E. coli* isolates susceptible to antimicrobial drug. TMP/SMX: trimethoprim/sulfamethoxazole; Ampi-Sulb: ampicillin-sulbactam; Pip/Tazo: piperacillin-tazobactam.

**Figure 3:**
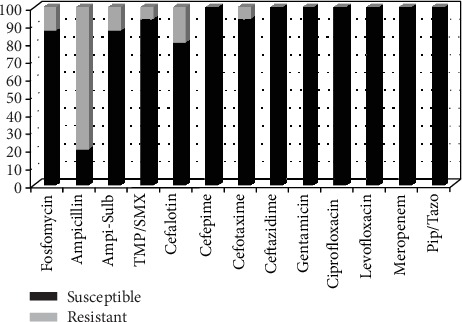
Percentage of *Klebsiella pneumoniae* isolates susceptible to antimicrobial drug. TMP/SMX: trimethoprim/sulfamethoxazole; Ampi-Sulb: ampicillin-sulbactam; Pip/Tazo: piperacillin-tazobactam.

Susceptibility testing showed that *E. coli*, including extended-spectrum beta-lactamase- (ESBL-) producing *E. coli*, was susceptible (100%) to fosfomycin (MIC ≤ 16 mg/L) and meropenem (MIC ≤ 0.25 mg/L). *E. coli* showed reduced susceptibility to ampicillin-sulbactam (Figure 2). The susceptibility patterns of *E coli* to ampicillin, TMP/SMX, and cefalotin were less than 60%. The susceptibility patterns of *K. pneumoniae* for 12 antibiotics tested in this study were more than 80%, except for ampicillin, which was less than 20% (Figure 3).

**Figure 4:**
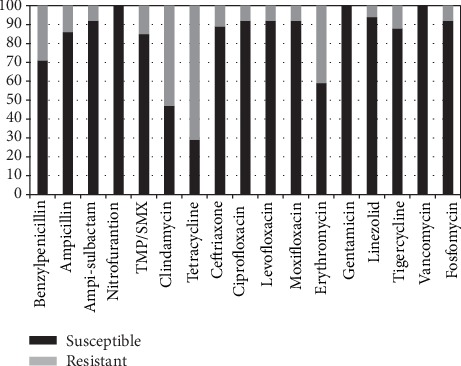
Percentage of Gram-positive bacteria isolates susceptible to antimicrobial drug. TMP/SMX: trimethoprim/sulfamethoxazole.

The susceptibility patterns of Gram-positive bacteria are shown in Figure 4. Susceptibility testing showed that a greater percentage of Gram-positive than Gram-negative bacteria was susceptible (more than 80%) to 13 antibiotics: nitrofurantoin, fosfomycin, ampicillin-sulbactam, ceftriaxone, ciprofloxacin, levofloxacin, moxifloxacin, gentamicin, vancomycin, linezolid, tigecycline, ampicillin, and TMP/SMX.

**Figure 5:**
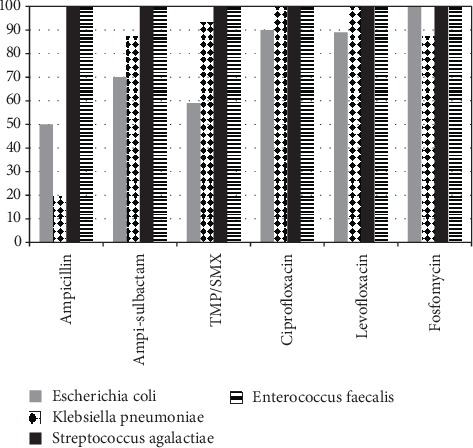
Susceptibility pattern of *E.coli*, *K. pneumoniae*, *S. agalactiae*, *E. faecalis* to antimicrobial drugs. Ampi-sulbactam: ampicillin-sulbactam; TMP/SMX: trimethoprim/sulfamethoxazole.

Figure 5 shows the percentage susceptibility of *E. coli*, *K. pneumoniae*, *S. agalactiae*, and *Enterococcus faecalis* to the antimicrobial drugs used to treat UTI. Fosfomycin and quinolone showed excellent susceptibility patterns for both Gram-positive and Gram-negative bacteria. Although quinolone had excellent susceptibility in vitro, it is not recommended for treating asymptomatic bacteriuria in pregnant women.

## 4. Discussion

Asymptomatic bacteriuria is common during pregnancy. It is a condition in which the urine culture reveals a significant growth of pathogens greater than 100,000 CFU/mL but without symptoms of UTIs. In this study, the prevalence of asymptomatic bacteriuria in pregnant women was 10.2%, slightly higher than the figure reported by the Indonesian Urogynecology Association of 2%–10% [12]. This was also slightly higher than the 10.0% reported in Thailand [14], 7.3% reported in Ghana [15], 4.1% reported in Brunei [16], and 3.6% reported in Sri Lanka [17]. It was, however, lower than the 29.5% reported in Nigeria [18] and the 30.5% reported in India [19].

Out of the 715 samples of pregnant women examined for nitrite/LE using the dipstick test, 135 showed positive results, yielding a prevalence of 18.9%. Of the 135 pregnant women with positive results, 73 (54.1%) had positive findings on urine culture. Although positive findings on nitrite and/or LE tests are often used as evidence of empirical antibiotic administration for asymptomatic bacteriuria in pregnancy to avoid several complications, such as preterm labor, low birth weight, preeclampsia, hypertension, renal failure, and intrauterine fetal death, culture is still needed to confirm the findings.

Based on the European Association of Urology (EAU) guidelines, some antibiotics can be administered to eradicate asymptomatic bacteriuria during pregnancy, including fosfomycin 3 g single dose; co-amoxicillin/clavulanate 500 mg q12h, 3–5 days; trimethoprim q12h, 3–5 days; amoxicillin 500 mg q8h, 3–5 days; cephalexin 500 mg q8h, 3–5 days; or nitrofurantoin 100 mg q12h, 3–5 days. In this study, the 135 pregnant women who had positive nitrite and/or LE test findings were given the option to choose fosfomycin-trometamol 3 g single-dose sachet or ampicillin/sulbactam. Out of the 76 pregnant women who took fosfomycin, two reported side effects of diarrhea that subsided without medication and fever that responded to paracetamol. Neonatal outcome was 100% full-term and normal-weight babies.

Empirical treatment guidelines need surveillance data on microbial patterns and antibiotic resistance. In this study, out of the 75 isolates, *E. coli* was found to be the most frequent causative organism (26.7%), followed by *K. pneumoniae* (20%). Similar findings have been reported from India [19–21] and Egypt [22], where the most common causative agent of asymptomatic bacteriuria in pregnant women was *E. coli* followed by the *Klebsiella* species. In contrast, in Brunei [16], the most common organisms were the *Klebsiella* species followed by *E. coli*. Although Gram-positive bacteria were found to be an etiological factor in this study, Gram-negative bacteria were found more common in cases of asymptomatic bacteriuria in pregnant women.

The susceptibility pattern of *E. coli*, including ESBL-producing *E. coli*, was 100% to fosfomycin and meropenem, and more than 80% of the isolates were susceptible to gentamicin, cefepime, cefotaxime, ceftazidime, ciprofloxacin, levofloxacin, and piperacillin-tazobactam. The use of ampicillin sulbactam as an empiric therapy for asymptomatic bacteriuria in pregnant women, a common occurrence in obstetric clinics in Indonesia, must be reconsidered because decreased susceptibility of *E. coli* to ampicillin-sulbactam was found in this study. The susceptibility of *E. coli* to ampicillin, TMP/SMX, and cefalotin was less than 60%. These results indicate that the use of TMP/SMX, ampicillin, and cefalotin is not an appropriate empiric therapy for asymptomatic bacteriuria in pregnant women.

The susceptibility of *K. pneumoniae* to the 13 antibiotics used in this study was more than 80%, including to fosfomycin, which was used as an empiric therapy in this study, and to ampicillin sulbactam, as an empiric therapy for asymptomatic bacteriuria in pregnant women. The susceptibility of *K. pneumoniae* to ampicillin was found to be less than 20%.

In this study, Gram-positive bacteria showed better susceptibility patterns than Gram-negative bacteria. The susceptibility of Gram-positive bacteria was 100% to nitrofurantoin, gentamicin, and vancomycin and more than 80% to fosfomycin, ampicillin-sulbactam, ampicillin, ceftriaxone, ciprofloxacin, levofloxacin, moxifloxacin, TMP/SMX, linezolid, and tigecycline. All Gram-positive bacteria were susceptible to nitrofurantoin. Although nitrofurantoin has been a long-standing choice for the treatment of uncomplicated UTIs such as asymptomatic bacteriuria in primary care settings because it is relatively cheap and the prevalence of resistance is generally acceptable, this antimicrobial drug is not available in Indonesia. Our findings indicate that appropriate empiric treatment for asymptomatic bacteriuria in pregnant women with affordable oral antimicrobial drugs is not readily available in Indonesia.

The common causative organisms of asymptomatic bacteriuria in this study such as *E. coli*, *K. pneumoniae*, *S. agalactiae*, and *E. faecalis* showed more than 80% susceptibility to fosfomycin, ciprofloxacin, and levofloxacin. Both Gram-positive and Gram-negative bacteria showed high susceptibility to fosfomycin and quinolone. Although quinolone had excellent susceptibility in vitro, it is not used to treat asymptomatic bacteriuria in pregnant women. Ampicillin-sulbactam and TMP/SMX can still be recommended as empiric therapy for asymptomatic bacteriuria in pregnant women, keeping in mind the reduced susceptibility of *E. coli*. Based on the EAU guidelines, TMP/SMX cannot be proscribed in the first trimester of pregnancy owing to the reported risk of congenital anomalies [10, 23].

This study clearly demonstrates the urgent need for periodic surveys to determine the susceptibility patterns of microorganisms that cause asymptomatic bacteriuria in pregnant women. Despite positive findings on the nitrite and/or LE tests, only 54.1% of the urine samples showed positive culture; therefore, culture results must be used to confirm asymptomatic bacteriuria and antimicrobial therapy should be based on the local pathogens' susceptibility pattern. Further, Gram-negative and Gram-positive bacteria have different susceptibility patterns.

Effective health policies need to be designed to reduce inappropriate use of antimicrobial drugs and to provide up-to-date guidelines on empiric treatment of uncomplicated UTIs, including asymptomatic bacteriuria, in pregnant women in Indonesia. To achieve better health outcomes and prevent complications and bacterial resistance, patient compliance to medication prescriptions is also very important.

## 5. Conclusion

Effective strategies for rapid and frequent surveillance are urgently needed to monitor antimicrobial resistance prevalent in low- and middle-income countries, where antimicrobial resistance is reaching unmanageable scales. Nitrite/LE test results, often used as evidence for empirical antibiotic administration for treating asymptomatic bacteriuria in pregnancy, should be confirmed using culture tests. The susceptibility pattern of Gram-negative and Gram-positive bacteria isolated from samples of asymptomatic bacteriuria patients in this study was more than 80% to fosfomycin, ciprofloxacin, and levofloxacin. *E. coli*, the most common causative organism of UTIs, including extended-spectrum beta-lactamase- (ESBL-) producing *E. coli*, showed 100% susceptible to fosfomycin. Therefore, the use of fosfomycin-trometamol is acceptable, based on in vitro susceptibility tests; it is safe for the treatment of asymptomatic bacteriuria in pregnancy, particularly for noncompliant patients. This result supports the guidelines of the IDSA, EAU, ESCMID, and Indonesian Society of Obstetrics and Gynecology on the use of fosfomycin as one of the primary medications for uncomplicated UTIs. However, mechanisms for prudent use and for monitoring bacterial susceptibility are needed to preserve this antibiotic for future use.

## Figures and Tables

**Table 1:** Characteristics of urine samples for enrolment of antibiotic susceptibility testing.

Community Health Centers	Number of samples		Trimester of pregnancy			Nitrite/LE		Fosfomycin	Bacteriuria ≥10^5^ CFU/mL	Gram	
	*N*	%	I	II	III	**+**	**−**			GN	GP
MKS	109	15.2	17	18	74	15	94	10	11 + 2	9	4
PGD	125	17.5	20	20	85	15	110	6	14	9	5
CAK	79	11.1	17	29	33	12	67	5	5	3	2
PSR	124	17.3	5	28	91	28	96	14	14	11	3
DSW	110	15.4	18	26	66	15	95	11	13	10	3
KJT	65	9.1	4	5	56	14	51	5	2	1	1
CIR	50	7.0	5	10	35	15	35	4	6	4	2
MTR	53	7.4	7	13	33	21	32	21	8	7	1
Total	715	100	93	149	473	135	580	76	75	54	21

Note: a single colony was obtained from most of the urine of pregnant women, but two of them had two bacterial isolates.

## Data Availability

The data in the tables used to support the findings of this study are included within the article.
